# Venomics Reveals the Venom Complexity of Sea Anemone *Heteractis magnifica*

**DOI:** 10.3390/md22020071

**Published:** 2024-01-28

**Authors:** Ming Li, Kailin Mao, Meiling Huang, Yanling Liao, Jinxing Fu, Kun Pan, Qiong Shi, Bingmiao Gao

**Affiliations:** 1 Key Laboratory of Tropical Translational Medicine of Ministry of Education, Hainan Key Laboratory for Research and Development of Tropical Herbs, School of Pharmacy, Hainan Medical University, Haikou 571199, China; mingli@hainmc.edu.cn (M.L.); maokailin@hainmc.edu.cn (K.M.); mgling213@163.com (M.H.); liaoyanling@hainmc.edu.cn (Y.L.); fjx1900201062@163.com (J.F.); pankun001219@163.com (K.P.); 2 Laboratory of Aquatic Genomics, College of Life Sciences and Oceanography, Shenzhen University, Shenzhen 518057, China; 3 Shenzhen Key Laboratory of Marine Genomics, Guangdong Provincial Key Laboratory of Molecular Breeding in Marine Economic Animals, BGI Academy of Marine Sciences, BGI Marine, Shenzhen 518081, China

**Keywords:** sea anemone, *Heteractis magnifica*, transcriptomics, proteomics, venom, toxin

## Abstract

The venoms of various sea anemones are rich in diverse toxins, which usually play a dual role in capturing prey and deterring predators. However, the complex components of such venoms have not been well known yet. Here, venomics of integrating transcriptomic and proteomic technologies was applied for the first time to identify putative protein and peptide toxins from different tissues of the representative sea anemone, *Heteractis magnifica*. The transcriptomic analysis of *H. magnifica* identified 728 putative toxin sequences, including 442 and 381 from the tentacles and the column, respectively, and they were assigned to 68 gene superfamilies. The proteomic analysis confirmed 101 protein and peptide toxins in the venom, including 91 in the tentacles and 39 in the column. The integrated venomics also confirmed that some toxins such as the ShK-like peptides and defensins are co-expressed in both the tentacles and the column. Meanwhile, a homology analysis was conducted to predict the three-dimensional structures and potential activity of seven representative toxins. Altogether, this venomics study revealed the venom complexity of *H. magnifica,* which will help deepen our understanding of cnidarian toxins, thereby supporting the in-depth development of valuable marine drugs.

## 1. Introduction

Like other cnidarians, sea anemones release venom through specialized cells known as nematocysts, which are hollow tubular structures containing numerous toxins [1]. Upon contact with prey, the tubule undergoes an explosive eversion, penetrating the target organism and releasing its venom. Among Cnidaria, sea anemones (Actiniaria) represent one of the most extensively studied groups with diverse toxins. In 1903, physiologist Charles Richet conducted pioneering research on *Actinia equina* and *Anemonia sulcata*, where he undertook the partial purification and characterization of two active components from the tentacular extracts [2]. Even though his research methods and subsequent chromatography made it possible to separate sea anemone toxins, most marine toxicologists still studied crude extracts of whole animals or antennae. Until the 1970s, *Anemonia sulcata* was homogenized and extracted with ethanol to isolate two new toxins ATX I and ATX II [3,4,5]. Later, scientists began to study other species of sea anemones. According to literature and database searches, 14 peptide toxins have been isolated from *H. magnifica* (HM) using traditional isolation and molecular cloning methods, including voltage-gated channel toxins HmK, Rp II, Rp III, Hmg 1b-2 and Hmg 1b-5 [6,7,8,9,10,11], protease inhibitor toxins HMIQ3c1, HMGS1, HMGS2 and HMRG1 [12], pore-forming toxins HMg I, HMg II, HMg III, and HmT, and hemolytic toxins [13,14,15,16]. However, access to different databases shows that the number of recognized toxins in *H. magnifica* is still relatively far from enough.

Although the scientific community has devoted considerable attention and conducted extensive research on sea anemone toxins, the identification of individual toxins has proven challenging. This difficulty primarily arises from the substantial venom sample required for isolation and characterization using classical biochemical techniques as well as the intrinsic instability of sea anemone’s venom proteins. The transcriptomic analysis of *Nematostella vectensis* was published in 2007 and marked the inspired beginning of an ‘omics’ era for sea anemone venom research [17]. This is the first time that transcriptome sequencing has been applied to sea anemones. So far, 16 sea anemones including *Cryptodendrum adhaesivum*, *Heterodactyla hemprichii*, *Heteractis crispa*, *Macrodactyla doreensis*, *Entacmaea quadricolor*, *Telmatactis stephensoni*, *Oulactis* sp, *Anemonia sulcata*, *Megalactis griffithsi*, *Exaiptasia diaphana*, *Pachycerianthus* cf, *Isarachnanthus nocturnus*, *Nematostella vectensis*, *Ceriantheomorphe brasiliensis*, *Pachycerianthus borealis* and *Stichodactyla helianthus* have been subjected to transcriptome sequencing [18,19,20,21]. The integration of transcriptome research into the investigation of sea anemone toxins has, to a certain extent, addressed the challenges associated with acquiring these venomous substances. However, owing to the scarcity of available reference genomes for cnidarians, the majority of these studies have adopted a strategy involving de novo transcriptome assembly from short reads. Therefore, transcriptome sequencing may produce false positive results that are difficult to distinguish. Recently, with the improvement in mass spectrometry instrumentation (for protein sequencing) and bioinformatic tools, proteomics has been used to identify sea anemone toxins. According to the literature, two sea anemones including *Bunodactis verrucosa* and *Nematostella vectensis* were subjected to proteomic sequencing [22,23]. In addition, some of the sea anemone toxins have been studied by venomics of integrating transcriptomic and proteomic technologies [24]. At present, three sea anemones (*Stichodactyla haddoni*, *Anthopleura dowii* and *Bunodosoma caissarum*) have been identified using integrated multi-omics technology for peptide discovery [24,25,26]. This approach can be complemented by the additional proteomic characterization of venom proteins, where the utilization of species-specific transcriptome annotation enhances protein identifications in comparison to searches in public databases [24,26]. These omics provide a detailed overview of the proteins and peptide toxins in sea anemones, revealing the high complexity and diversity of venom compared to traditional research results [19].

Many venomous species possess a central venom gland or duct through which toxic peptides and other biomolecules can be injected into predators and prey [27,28]. Cnidarians stand out as one of the few organisms without a centralized venom transmission system. Instead, their venom is transmitted at the cellular level through specialized stinging capsules known as nematocysts. In sea anemones, these nematocysts are distributed in various regions of polyps, including the actinopharynx, tentacles, column, acrorhagi, and mesenterial filaments. Attributed to differences in various physiological functions, there are also differences in the venom produced by nematode vesicles between tissues [29], providing more opportunities to regulate the composition of the venom and allowing the same organism to produce multiple types of venom [30]. 

Sea anemones lack active locomotion ability, and during their evolution, they choose to rely on toxins in their tentacles to cause paralysis, thereby fixing food and repelling predators. Therefore, the nematocyst in tentacles is the most abundant tissue and serves as the primary source for extracting toxin peptides [31,32,33]. In addition, the mesenterial filaments of sea anemones house venom components to play a crucial role in aiding digestion. However, these filaments can also be projected beyond the anemone’s body, serving functions in external digestion, competition, and defense [29]. Accumulated evidence suggests that various species within the order Actiniaria (sea anemones) exhibit a differential expression of toxins during distinct life stages and in different anatomical regions [34,35]. In tentacles, mesenterial, filaments, and the body column, different toxin profiles have been observed [35]. However, there is currently no transcriptomic or proteomic study on the distribution, diversity, and potential medicinal value of peptide toxins in different tissues of *H. magnifica*, including tentacles and the column.

In this work, in order to explore the complexity and diversity of toxins in *H. magnifica*, integrated venomics including transcriptomics and proteomics sequencing was applied to study the tentacles and column of this sea anemone species. These findings will contribute to the expanding repertoire of *H. magnifica* venom proteins and peptides as well as aid in designing targeted experiments for further isolation and characterization of more valuable specific peptides. Moreover, the identification of putative venom peptides will offer new insights into the evolutionary diversification of toxins and various strategies for prey capture or predator defense.

## 2. Results

### 2.1. Summary of the Transcriptome Sequencing and Assembly

**Figure 1:**
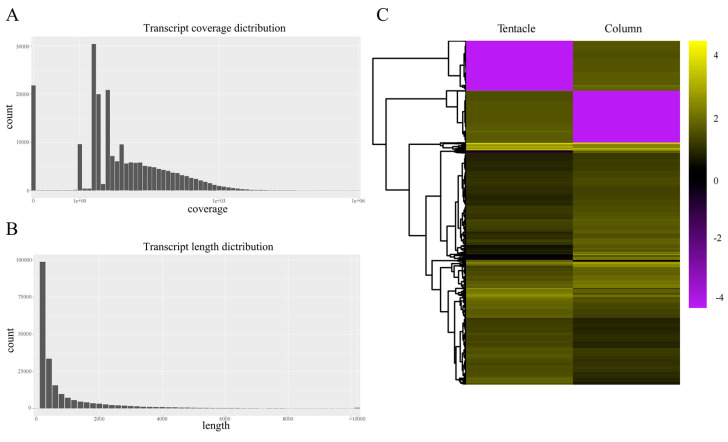
Summary of the *H. magnifica* transcriptome assembly. (**A**) The coverage of assembled transcripts after mapping of raw sequences back to the assembly; (**B**) the transcript length distribution; (**C**) a heatmap of differentially expressed genes (DEGs; colored for RPKM values) for the column and tentacles tissues in *H. magnifica*.

A total of 41,408,576 and 44,188,792 reads were generated using an Illumina sequencing platform for the tentacles (HM-T) and column (HM-C), respectively (Supplementary Table S1). After the removal of low-quality reads, we selected 207,530 clean reads for the tentacles (with an average length of 942 bp) and 148,422 clean reads for the column (with an average length of 900 bp) for further assembly and mapped the original sequences back to the assembled transcripts (Figure 1A). These tentacles and column short reads were then de novo assembled into 134,263 and 94,024 transcripts, respectively, by paired-end joining and gap filling with the Trinity software [36,37].

The average length of tentacles transcripts was 675 bp with an N50 of 1105 bp, and the average length of the column transcripts was 721 bp with an N50 of 1256 bp (Figure 1B and Supplementary Table S1). Gene transcription in *H. magnifica* was investigated through tissue-specific transcriptome sequencing (RNA-seq) conducted on two anatomically distinct regions including the tentacles and column. To estimate the relative abundance of each transcript, the paired trimmed reads were mapped back to the transcriptome assembly. The values of Fragments Per Kilobase of the exon model per Million mapped fragments (FPKMs) were then calculated using Stringtie (v1.3.3 b, Mihaela Pertea laboratory, Baltimore, MD, USA). Differential gene expression was assessed through tissue-specific RNA-seq in the two anatomically distinct regions (FDR ≤ 0.05) (Figure 1C).

### 2.2. Statistics of Transcriptome Annotation

To assess the functions of assembled transcripts, a total of 134,263 unigenes from the tentacles and 94,024 unigenes from the column were functionally annotated based on five public databases. These annotations predicted 44,831 genes in the tentacles (Supplementary Figure S1 and Supplementary Table S2) and 29,174 genes in the column (Supplementary Figure S2 and Supplementary Table S3). Among the annotated 44,831 tentacles genes, approximately 18,370 genes were categorized into five main processes (environmental information processing, cellular processes, genetic information processing, organic systems, and metabolism) according to the KEGG analysis (Supplementary Figure S3). The KOG database identified and annotated 17,778 genes, classifying them into 25 molecular families (Supplementary Figure S4). Moreover, the GO annotation revealed that 25,217 genes were associated with 29 functional annotations, which were further categorized into three distinct biological processes (Supplementary Figure S5). 

Out of the 29,174 genes identified in the column, 11,964 genes were further classified into five primary categories (environmental information processing, cellular processes, genetic information processing, organic systems, and metabolism; see Supplementary Figure S6). A total of 11,868 unigenes were annotated with KOG classifications and grouped into 25 molecular families (Supplementary Figure S7). Additionally, another GO analysis revealed that 17,458 unigenes were associated with 29 functional annotations, which were further divided into three distinct biological processes (Supplementary Figure S8). These findings indicated that over 79.14% and 86.42% of reads from the tentacles (Supplementary Figure S9) and column (Supplementary Figure S10), respectively, corresponded to some sea anemones, with *Actinia tenebrosa* and *Exaiptasia diaphana* as the top two matched species. The annotated genes from the tentacles (44,831) and column (29,174) were deduced as reference protein databases for subsequent proteomic studies.

### 2.3. Toxins Identified in the Transcriptomes of H. magnifica

**Figure 2:**
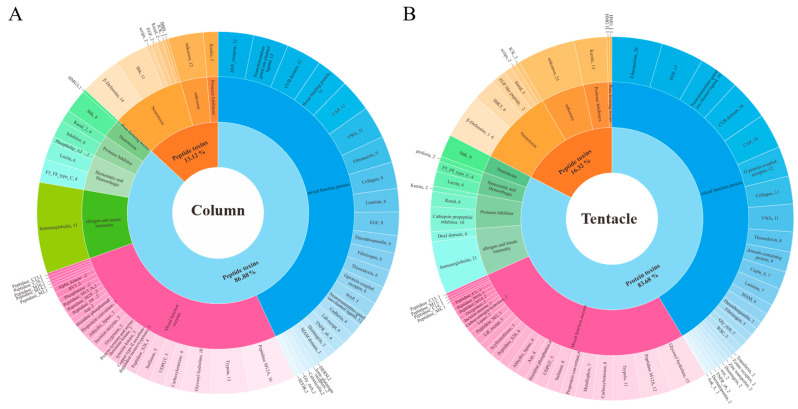
Putative toxin families identified in the transcriptomes of *H. magnifica*. The distinctive protein and peptide sequences from the tentacles (442) and the column (381) were compared by blasting the UniProt database (www.UniProt.org/program/toxins, Accessed on: 30 August 2023). They were then classified into toxin families based on their cysteine scaffolds and amino acid sequences. The pie charts illustrate the proportional contribution of each toxin family to the predicted venom proteome. The number of homologs identified for each protein and peptide family is displayed next to the family name. (**A**) Classification of the tentacles transcriptome; (**B**) classification of the column transcriptome.

To identify candidate toxins in *H. magnifica*, the assembled transcripts were compared to the UniProt animal toxin database using BLAST [18]. A total of 630 protein sequences and 98 peptide sequences were identified in transcriptomics. A total of 442 sequences were identified in tentacles, of which 367 were proteins and 75 were peptides. Out of 381 sequences in the column, 332 protein sequences and 75 peptide sequences were identified (Figure 2). These transcripts were divided into 68 groups according to the predicted functions and then categorized into various biological functions, such as neurotoxin, hemostatic and hemorrhagic toxins, pore-forming protein, mixed-function enzymes, protease inhibitors, allergen, and innate immunity, and mixed-function protein (Figure 2).

**Figure 3:**
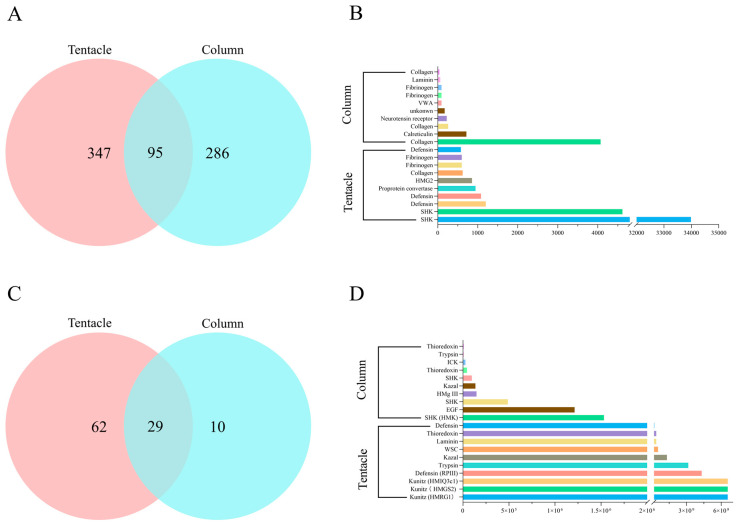
Comparison of proteome and transcriptome data in the *H. magnifica* tissues. (**A**) Relationship between the tentacles and the column data from the transcriptome datasets of *H. magnifica*; (**B**) ten transcripts of proteins and peptides with the highest levels of transcription from the *H. magnifica* transcriptomes; (**C**) relationship between the tentacles and the column data from the proteomic datasets of *H. magnifica*; (**D**) ten proteins and peptides with the highest levels of translation from the *H. magnifica* proteomes.

The tentacles and column transcriptomes of *H. magnifica* were predicted with 442 and 381 toxin transcripts, respectively (Figure 3A and Supplementary Tables S4 and S5). In addition, this experiment also used FPKM values to represent the transcription levels of the genes encoding proteins or peptide toxins to evaluate the differential expression sequences between different tissues, including tentacles and columns. The top 10 protein or peptide toxins, with the highest FPKM values, were selected from each dataset (Figure 3B). The results showed that ShK-like peptides are the most abundant toxin transcripts in the tentacles, while collagen is the most abundant toxin transcript in the column. The tentacles of *H. magnifica* contained a significantly higher transcription of ShK-like peptides compared to the column. As one of the most crucial toxins present in sea anemone venoms, ShK-like peptides usually play a critical role in protecting sea anemones during defense and predation activities [38]. Furthermore, the collagen in the column exhibited a relatively higher transcription level compared to the tentacles.

### 2.4. Proteomic Analysis of H. magnifica Venom Proteins and Peptides

A total of 101 proteins and peptides were identified in the tentacles and column samples, of which 79 were protein sequences and 22 were peptide sequences, with the assistance of a local protein database generated by TransDecoder from our transcriptome data of the *H. magnifica* tentacles and column (Supplementary Table S6). 

These identified proteins and peptides were further classified by family, class, or main molecular function according to the UniProt database and then grouped into seven main categories. They included 36 functional proteins, 26 proteases, 23 neurotoxins, 9 protease inhibitors, 5 allergen and innate immunity proteins, 1 pore-forming toxin, and 1 hemostatic and hemorrhagic protein. Among them, 91 were identified in the tentacles, and 39 were present in the column (Figure 3C). Furthermore, among the 29 co-expressed sequences, 22 were peptide toxins and 7 were protein toxins (Supplementary Table S6). The comparison of transcriptome and proteome expression levels showed significant variances in the highest expressed proteins. The proteome analysis showed that those peptides with the highest expression levels in the tentacles were HMRG1, HMGS2, HMIQ3c1, and RPIII, which have been studied in previous reports [6,12]. HmK, HMg III, and EGF-like peptides are highly expressed in the column. HM-052 is the same sequence as the reported HmK, and HM-028 is an EGF-like peptide with high similarity to Gigantoxin I (Figure 3D). The proteomic analysis detected a considerable abundance of toxins linked to various types, including Kunitz-type peptides, Anemone type 5 potassium channel toxins, ShK-like peptides, and β-defensin peptides. These toxin sequences also showed significant similarities to previously documented members of corresponding families (Supplementary Table S6).

#### 2.4.1. ShK-like Peptides

**Figure 4:**
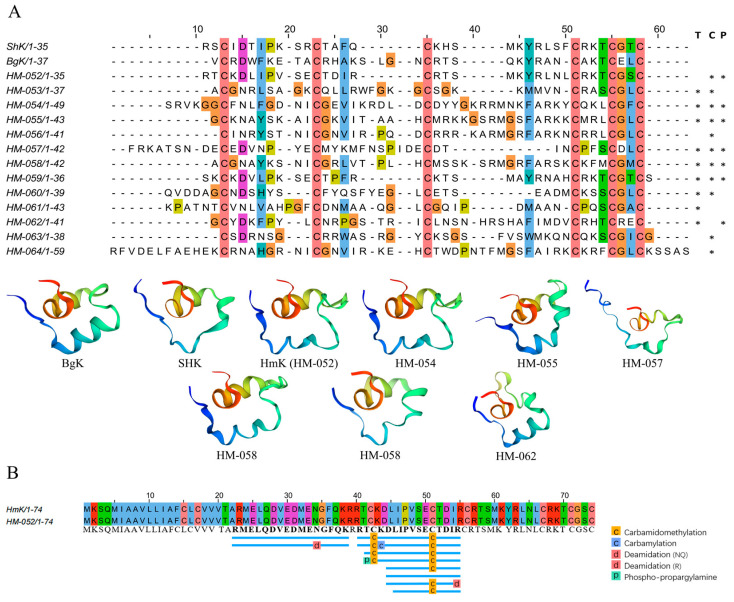
Deduced ShK domain transcripts from *H. magnifica*. (**A**) Peptide sequences from the mature region of representative sea anemones with the ShK domain were aligned for comparison. Residue shading was highlighted based on the Clustal color scheme (T: tentacles transcriptome, C: column transcriptome, P: proteomics; *: identification of sequences in corresponding omics data); (**B**) Peptide coverage.

ShK-like peptides are important toxins in sea anemones, and 13 ShK-like peptides were identified in the transcriptomes of *H. magnifica*. Subsequent proteomic analysis confirmed seven of these sequences, with HM-052 being exclusively expressed in the column and HM-062 only in the tentacles. The remaining five sequences were co-expressed in both the tentacles and the column (Figure 4A). Additionally, the transcriptomic analysis revealed 14 proteins containing ShK-like peptides, and the proteomic validation confirmed five of these proteins (Supplementary Table S6). Notably, HM-701 and HM-703 were uniquely expressed in the tentacles, while the remaining ShK-like peptides were identified in both the tentacles and the column (Supplementary Table S6). The results also showed that HM-052 has been reported in the literature as HmK, which is the first ShK-like peptide discovered in *H. magnifica* [39] (Figure 4B). 

#### 2.4.2. β-Defensin Peptides

β-defensin peptides, secreted as components of the innate immune system in various organisms, are one of the best-described groups of antimicrobial peptides from a large number of plants, animals, and fungi for host defense [40,41]. However, the β-defensin peptides subfamily has been converted into neurotoxins in several animals to block ion channels [42]. There are 23 β-defensin peptides in the transcriptome, of which nine are only identified in tentacles, seven are only identified in column, and seven are co-expressed.

**Figure 5:**
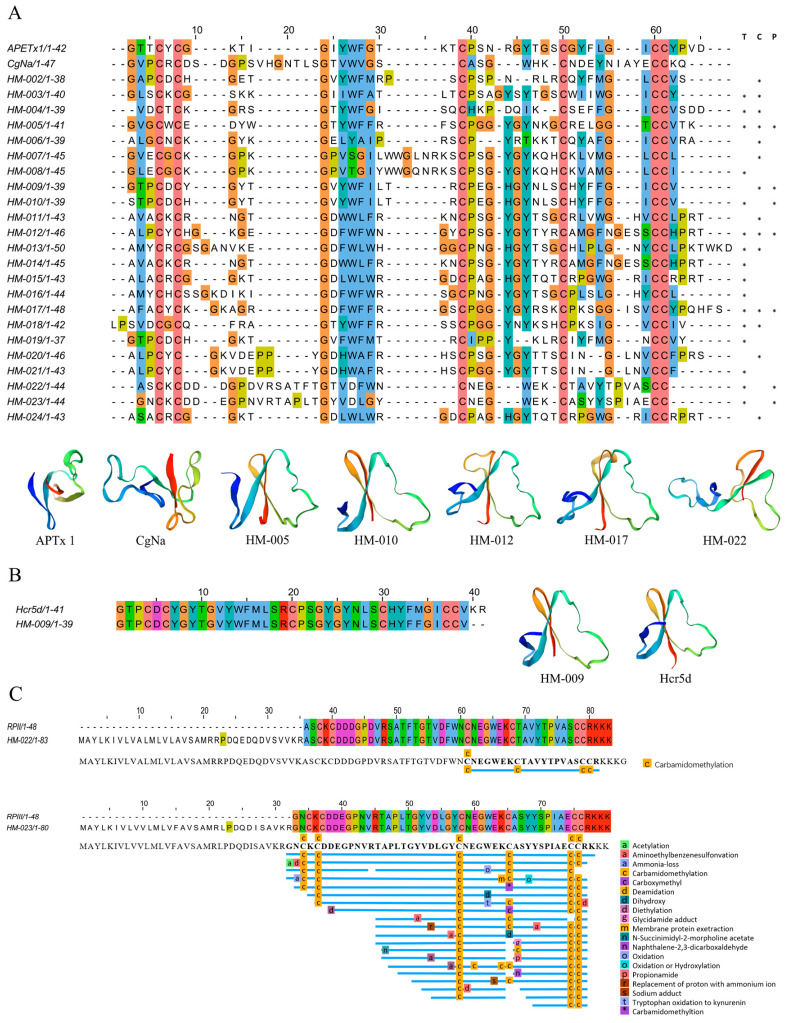
Deduced β-defensin transcripts from *H. magnifica*. (**A**) Representative mature region peptide sequences with β-defensins. These sequences were aligned using MUSCLE and visualized with Jalview, employing residue shading based on the Clustal protein color scheme (T: tentacles transcriptome, C: column transcriptome, P: proteomics; *: Identification of sequences in corresponding omics data); (**B**) similar to a sequence from *Heteractis crispa* (Hc) in transcriptome sequence alignment; (**C**) peptide coverage.

Among them, seven transcript sequences belonging to β-defensin peptides were confirmed in the proteome data using the NCBI blast tool (Supplementary Table S6). Specifically, HM-022 and HM-023 were identical to the reported toxins RP II and RP III, respectively (Figure 5A,C). These toxins can inhibit signal transduction during the inactivation of sodium channels. Furthermore, HM-009 identified by the proteome showed a high similarity (97%) with Pi-shtx-Hcr5d obtained by Kalina et al. from *Heteractis crispa* (Figure 5B) [43], and it showed a similarity (87%) with toxin Hmg 1b-5 from *H. magnifica*.

#### 2.4.3. Inhibitor Cystine Knot Fold Peptides

**Figure 6:**
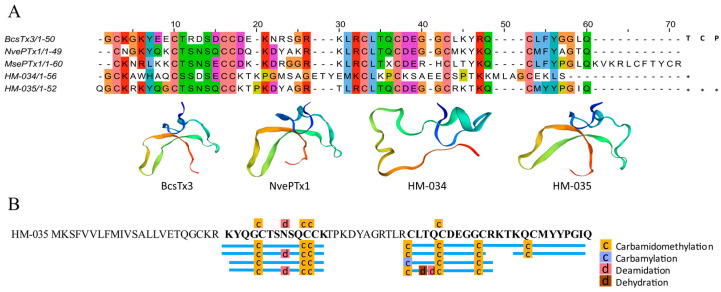
Deduced ICK transcripts from *H. magnifica*. (**A**) Representative peptide sequences with ICK domains. Sequences were aligned with residue shading, which is based on the Clustal protein color scheme (T: tentacles transcriptome, C: column transcriptome, P: proteomics; *: identification of sequences in corresponding omics data); (**B**) peptide coverage.

Through transcriptomic studies, two inhibitor cystine knot fold (ICK) peptides were successfully identified, namely HM-034 and HM-035 (Figure 6A). HM-034 was only specifically identified in the tentacles transcriptome. It is worth noting that the HM-035 has been detected in both the tentacles and the column, and its existence has been confirmed by the proteomic analysis (Figure 6B and Supplementary Table S6). This cysteine pattern is also distinctive and aligns with only the voltage-gated potassium channel (Kv) type 5 toxin documented thus far, namely BcsTx3 (κ-actitoxin-Bcs4a) from *Bunodosoma caissarum* [44].

#### 2.4.4. EGF-like Peptides

**Figure 7:**
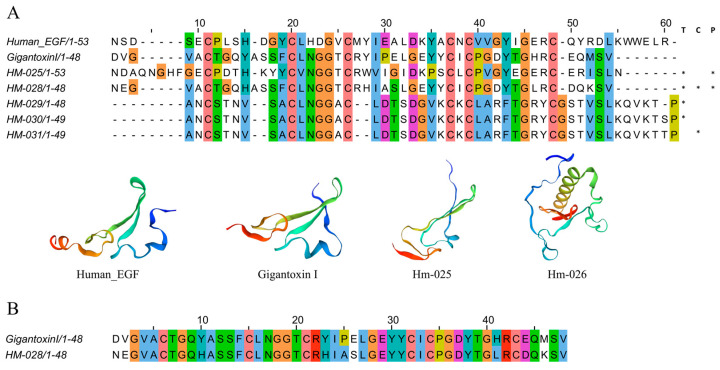
Deduced EGF domain transcripts from *H. magnifica*. (**A**) Representative mature peptide sequences with EGF domains. Residue shading was applied with the Clustal color scheme (T: tentacles transcriptome, C: column transcriptome, P: proteomics; *: identification of sequences in corresponding omics data); (**B**) similar sequence in *Stichodactyla gigantea*.

Several peptide factors bind to specific receptors on the cell surface to control cell growth, differentiation, and survival. Recent studies have revealed that diverse invertebrates also express a variety of growth factors and growth factor receptors [45]. By conducting a comprehensive transcriptome analysis of *H. magnifica*, a total of five peptide sequences (Figure 7A) and 23 protein sequences with EGF-like peptides were discovered (Supplementary Table S7). A further proteomic investigation supported the existence of three EGF-like peptide analogs and one EGF domain protein in the tentacles (Supplementary Table S6). Specifically, our experiments led to the identification of HM-025, HM-026, and HM-028 as EGF-like peptides, while the HM-028 exhibited a strikingly high sequence similarity (81%) with Gigantoxin I (Figure 7B). In fact, Gigantoxin I is one representative EGF-like peptide in various sea anemones; it was first isolated from *Stichodactyla gigantea*. Both EGF activity and toxicity are observed in this toxin, which causes tyrosine phosphorylation of the EGF receptor in A431 cells [46].

#### 2.4.5. Kunitz-Type Peptides

**Figure 8:**
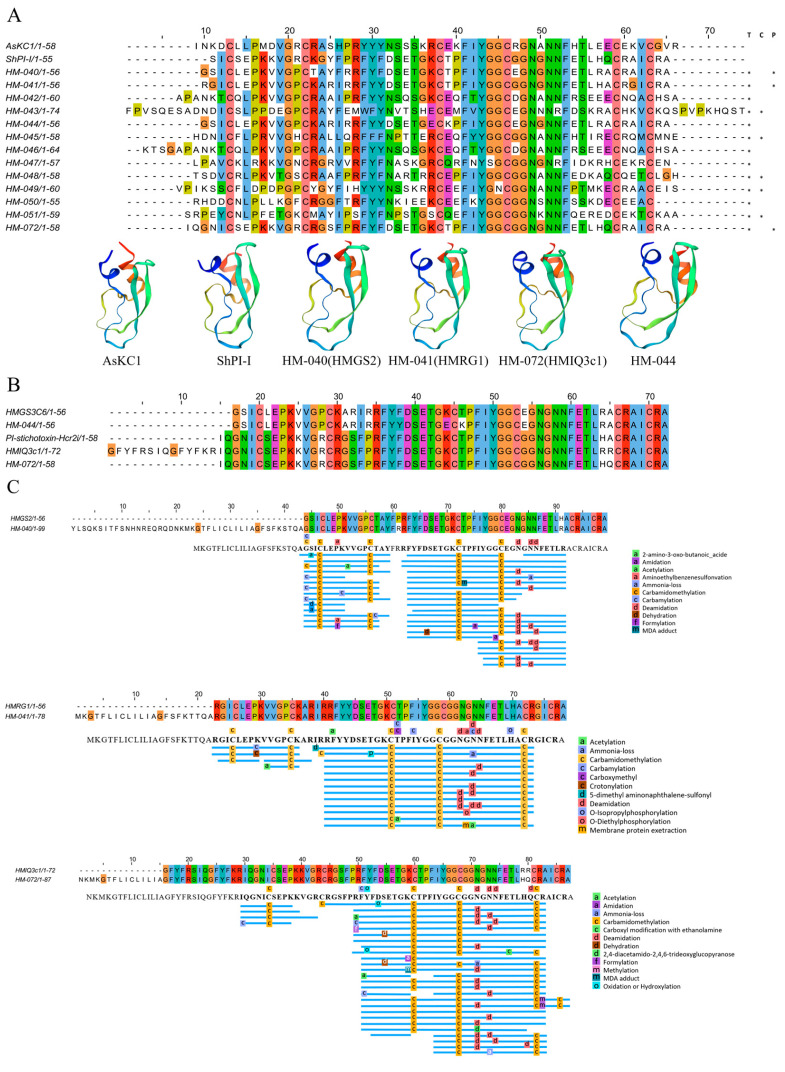
Deduced Kunitz-type transcripts from *H. magnifica*. (**A**) Representative mature peptide regions with Kunitz-type peptides. Residue shading was applied with the Clustal color scheme (T: tentacles transcriptome; C: column transcriptome, P: proteomics; *: identification of sequences in corresponding omics data); (**B**) similar sequence in the sea anemone *Heteractis crispa* (Hc); (**C**) peptide coverage.

The Kunitz-type protease inhibitors are widespread [47]. The transcriptomic analysis of *H. magnific* in this study revealed 12 homologous sequences, including eight peptides expressed in the tentacles and five sequences expressed in the column (Figure 8A), and the proteomic analysis supported the existence of three previously identified Kunitz-type peptides (Supplementary Table S6).

Specifically, HM-040 and HM-041 share the same sequence as HMGS2 and HMRG1 toxins (Figure 8C) that were previously reported [12]. HM-072 is very similar to the previously reported HMIQ3c1 [48] (Figure 8B) with only arginine substituted for histidine and glutamine at positions 50 and 51. In addition, the HM-044 sequence exhibits a high similarity (95%) with the reported toxin HMGS3c6 (Figure 8B). The main difference is that phenylalanine replaces tyrosine at position 22 on HM-044 (Figure 8B). During the 1970s, the first report of protease inhibitors was published in sea anemones [49,50]. Subsequent studies have yielded additional information on Kunitz-type peptides, such as AsKC1 and ShPI-I toxins derived from the anemones *Anemonia sulcata* and *Stichodactyla haddoni*, respectively [51,52]. Previous findings proved that Kunitz-type peptides have dual functions as potassium channel blockers and serine protease inhibitors, enabling them to act as defense molecules and neurotoxins for immobilizing prey or predators [53].

#### 2.4.6. Kazal Domain Peptides

**Figure 9:**
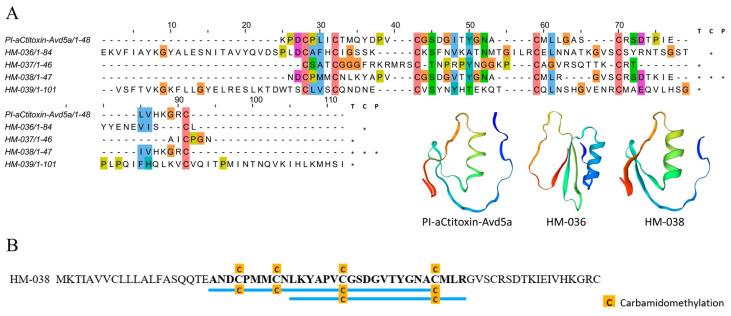
Deduced Kazal domain transcripts from *H. magnifica*. (**A**) Representative mature peptide sequences with Kazal domain peptides. Residue shading was applied with the Clustal color scheme (T: tentacles transcriptome, C: column transcriptome, P: proteomics; *: identification of sequences in corresponding omics data); (**B**) peptide coverage.

Kazal-type serine proteinase inhibitors (KPIs) are characterized by the presence of one or more Kazal domains [54]. Here, a total of 12 Kazal domain peptides were identified through transcriptomic analysis, and four peptide sequences HM-036 to HM-039 exhibited the same cysteine pattern as the previously reported toxin, PI-atitoxin-Avd5a (Figure 9A). Among them, only the peptide HM-038 and two proteins HM-723 and HM-724 containing Kazal domains were validated by the proteomic analysis (Figure 9B and Supplementary Table S6). The Kazal domain peptides produced by the glandular cells of sea anemones are reported to exhibit antibacterial activity against *Streptomyces aureus*; furthermore, they are closely associated with the immune defense response of sea anemones [55].

#### 2.4.7. Pore-Forming Toxins

**Figure 10:**
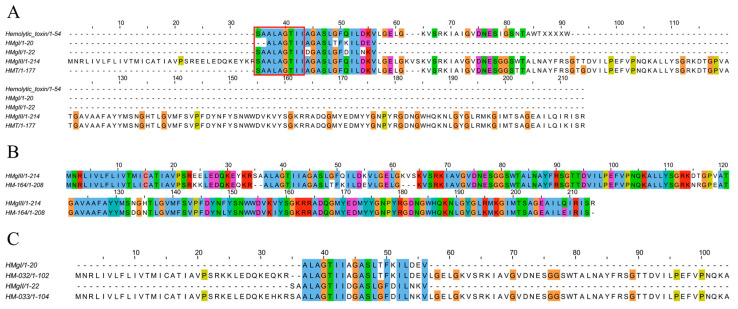
Deduced pore-forming transcripts from *H. magnifica*. (**A**) Representative sequences with pore-forming peptides. Residue shading was applied with the Clustal color scheme; (**B**) proteomics and transcriptomics identification of HMg III-like toxin; (**C**) transcriptomics identification of HMg I-like and HMg II-like toxins.

Pore-forming toxins (PFTs) are frequently reported components in cnidarian venoms [56]. Five PFTs including HMg I, HMg II, HMg III, HmT and hemolytic toxins have been reported in *H. magnifica* (Figure 10A) [13,14,15,16]. Here, three putative PFTs were identified in the tentacles transcriptome of *H. magnifica*. HM-164 was confirmed by proteomics, and its sequence is similar to HMg III. The peptides HM-032 and HM-033 are new pore-forming toxins. However, their results of sequence alignment showed that both peptides also contained HMg I and HMg II sequences, respectively (Figure 10B,C). 

## 3. Discussion

Here, we for the first time reported the integrated transcriptome and proteome sequencing of *H. magnifica*. The transcriptomic analysis identified 728 putative toxin sequences, including 442 and 381 from the tentacles and the column, respectively, and they were assigned to 68 gene superfamilies. More importantly, the subsequent proteomic analysis confirmed 101 protein and peptide toxins in the venom, including functional proteins (such as pore-forming proteins, collagens, and G protein-coupled receptors, neurotoxins such as ShK-like peptides, ICK peptides, and β-defensin peptides) and proteases (such as metalloproteinases, lipases, and other enzymes). 

ShK-like peptides were named after the first toxin identified in the sea anemone *Stichodactyla helianthus* [57], block Kv1.3 or Kv1.1 and suppressing the proliferation of effective memory T cells at picomolar concentrations [58,59,60]. Here, seven ShK-like peptides including HmK were identified in the tentacles and the column of *H. magnifica*, all of which were present in the transcriptome and proteomic data. These peptide toxins have therapeutic potential for autoimmune diseases involving effective memory T cells activated by Kv1.3 potassium channels [25]. Eventually, ShK-186 was developed, which was 100 times more selective for Kv1.3 than for Kv1.1, Kv1.4 and Kv1.6 [61,62,63]. These ShK-like peptides have not been reported in the venom proteomes of other sea anemone species. Therefore, the ShK-like peptide library from various sea anemones may be more diverse beyond our expectation. In addition, the proteomic results showed that ShK-like peptides are highly expressed in the column, and the column usually recognizes toxins more than tentacles. Previous studies have shown that the interstitial filaments in sea anemones contain venomous components to aid digestion, but they may also be projected outside the sea anemone for different purposes, such as external digestion, competition, and defense [64].

The most abundant toxin family identified in this study was β-defensin peptides with 22 transcriptome sequences, seven of which were validated by the proteomics analysis. These peptide members share a defensin domain. Two peptides matched the previously described toxins from *H. magnifica* (Rp II and Rp III), which can selectively inhibit voltage-gated sodium channels according to the UniProt database. β-defensin peptides are often crucial antimicrobial peptides, which are secreted as integral components of the innate immune system in various organisms [65,66]. In the sea anemone venoms, β-defensin peptides exhibit potential neurotoxic properties by blocking ligands and voltage-gated ion channels, including voltage-gated sodium channel (NaV) types 1, 2, and 4, Kv type 3, and acid-sensing ion channels (ASIC) [67,68,69,70]. Our multi-omics results show that more β-defensin peptides are recognized by tentacles, and the β-defensin peptides in sea anemone venoms may have evolved for predation and defense purposes with the ability to induce pain and prevent potential predators [38].

The Kunitz-type peptides have a highly conserved region, being characterized by serine protease inhibition, which are also observed among various sea anemones. Three peptides with protease inhibitory activity, such as HMIQ3c1, HMGS2 and HMRG1, were identified in this study. The physiological role of these inhibitors may be related to resistance to prey proteases from degrading their venom peptides [71]. Some of them have shown inhibitory potential against proteases of other classes or the ability to block voltage-gated potassium ion channels, which are crucial regulators of various physiological processes such as host defense, blood coagulation, platelet modulation, fibrinolysis, and action potential transduction [72,73,74]. 

In the transcriptome data, many transcripts from the tentacles of *H. magnifica* showed a high percentage of identity with reported transcripts from some closely related species. By comparing different sea anemone toxins, HM-009 and HM-072 in *H. magnifica* transcriptomes are similar in sequence to the Hcr5d and Hcr2i from *Heteractis crispa*. This indicates that these peptide toxins with important roles may be conserved somehow in various sea anemones, although there are certain significant differences in the identified peptide toxins among different species. 

Transcriptomics is inherently constrained to identifying candidates with significant sequence homology to known toxins, and it is susceptible to generating false positives [75,76,77]. Therefore, in this study, proteomics was integrated to further verify the transcriptomic data of different tissues to obtain the complete venom composition. The significance of incorporating proteomic data becomes apparent when considering the discrepancy in both the number and type of putative toxin families during identification through our homology-based annotation compared to those findings derived from the combined transcriptome and proteome data. These data in this study reinforce the need to employ specific databases for further proteomics analyses in order to search for new peptide sequences with novel biological activities.

## 4. Materials and Methods

### 4.1. Sea Anemone Collection

Specimens of wild *H. magnifica*, 30–50 cm in length, were collected from offshore areas of Sanya City, Hainan Province, China. These specimens were kept alive in indoor aquaria with artificial seawater. After at least two weeks, they were transferred to smaller tanks for acclimation of 15 min, and then the tentacles and column tissues were cut using tweezers and a scalpel. Once these tissues were removed for storage in liquid nitrogen, the specimens were returned to their original aquarium for continuous aquaculture.

### 4.2. Transcriptome Construction and Quality Checking

For each tissue, total RNA was extracted using an RNeasy Mini Kit (Qiagen, Duesseldorf, North Rhine Westphalia, Germany). Then, the RNA extractions were quantified on a nanodrop spectrophotometer (Thermo Scientific, Waltham, MA, USA), and RNA integrity number (RIN) values were determined on an Agilent 4200 Bioanalyzer (Agilent, Santa Clara, CA, USA). Illumina sequencing libraries were constructed from total RNA using the NEBNext Ultra RNA Library Prep Kit (NEB, Beverly, MA, USA). The qualified library was sequenced on an Illumina Novaseq 6000 (Illumina, San Diego, CA, USA) high-throughput sequencing platform. The applied sequencing strategy is PE 150. Each sample contained at least 6 GB of sequencing data. Due to the importance of initial error correction on the transcriptome assembly, the initial data are screened for quality control. After filtration of those low-quality reads (containing more than 1% non-sequenced bases or more than 50% low-quality bases) as well as adapter sequences, paired-end sequences were de novo assembled into contigs using Trinity with default parameters [78,79].

### 4.3. Functional Annotation of the Assembled Transcriptome

To identify potential toxin-like transcripts, the translated transcriptome data were compared with putative animal toxin sequences, which were previously achieved by searching various public databases (with an E-value ≤ 10^−5^ as the threshold) [80]. Searches were conducted against five public databases, including (a) the Kyoto Encyclopedia of Genes and Genomes (KEGG) [81,82], (b) cnidarian protein sequences from the GenBank non-redundant (Nr) protein database [83], (c) UniProt animal toxin and venom database (UniProt) [84], (d) clusters of orthologous groups for eukaryotic complete genomes (KOG) [85], and (e) Gene Ontology (GO).

### 4.4. Candidate Toxin Gene Identification

A BLASTn (E-value < 10.0 and matching length > 60%) search was performed on the transcriptomes using toxin genes from cnidarians and other venomous lineages in the ToxProt dataset (http://www.uniprot.org/program/Toxins, accessed on 30 August 2023) and NCBI Nr Cnidarian Venomous Lineages to identify candidate toxin genes from the combined transcriptomes for each focal taxa [86]. Toxin-related genes from these sea anemone toxin candidates were recognized by SignalP 4.0 (http://www.cbs.dtu.dk/services/SignalP/, accessed on 15 September 2023) as signal peptides and mature peptide regions. Finally, only sequences containing the predicted signal peptides and mature peptides were considered as putative toxin candidates.

### 4.5. Identification of Protein and Peptide Toxins

A BLAST search of the UniProt database was performed manually to identify sea anemone proteins and peptide toxin families. Subsequent analysis and validation of the cysteine frameworks of peptides and protein toxins were conducted. Proteins and peptide toxins were assigned to known superfamilies in the BLAST database (in our present study, an amino acid number less than 80 was defined as “peptide”).

### 4.6. Venom Sample Preparation for the Proteomic Analysis

Fresh tentacles and column tissues from *H. magnifica* were collected. Sample preparation includes protein extraction, denaturation, reduction, and alkylation as well as tryptic digestion and peptide cleanup. A commercially available iST Sample Preparation kit (PreOmics, Planegg, Germany) was applied according to the manufacturer’s protocols. Briefly, 50 µL of Lyse buffer was added to each sample with heating to 95 °C for 10 min and rotation at 1000 rpm. After cooling down to room temperature, trypsin digestion buffer was added, and these samples were incubated at 37 °C for 2 h with shaking at 500 rpm. The digestion process was stopped with a stop buffer. Clean-up and desalting were carried out in the iST cartridge using the recommended washing buffers. Peptides were eluted with elution buffer (2 × 100 µL) and then dried in a speed vacuum concentrator.

### 4.7. Tandem Mass Spectrometry (MS/MS)

These peptide samples were subsequently re-dissolved in solvent A (0.1% formic acid in distilled water) and analyzed by Q-Exactive Plus coupled to an EASY-nanoLC 1200 system (Thermo Fisher Scientific, Waltham, MA, USA). Every 3 μL peptide sample was loaded onto a 25 cm analytical column (75 μm inner diameter, 1.9 μm resin (Dr. Maisch)) and separated with 60 min gradient starting at 2% buffer B (80% acetonitrile with 0.1% formic acid) followed by a stepwise increase to 35% in 47 min and then 100% in 1 min, which was held for 12 min. The column flow rate was maintained at 300 nL/min with the column temperature at 40 °C. The electrospray voltage was set to 2 kV. The mass spectrometer was run under a data-dependent acquisition (DDA) mode and automatically switched between MS and MS/MS modes. The survey of full-scan MS spectra (*m*/*z* 350–1800) was acquired in the Orbitrap with 70,000 resolutions. The automatic gain control (AGC) target of 3 e6 and the maximum injection time of 50 ms were set up. Then, the precursor ions were selected into the collision cell for fragmentation by higher-energy collision dissociation (HCD), and the normalized collection energy was set at 28. The MS/MS resolution was set at 17,500 with the automatic gain control (AGC) target of 1 × 10^5^, the maximum injection time of 45 ms, and dynamic exclusion for 30 s.

### 4.8. Spectral Searches and Bioinformatics Analysis

Tandem mass spectra were processed by PEAKS Studio version 10.6 (Bioinformatics Solutions Inc., Waterloo, ON, Canada). PEAKS DB was set up to search the database of targets assuming trypsin as the digestion enzyme. The DB was searched with a fragment ion mass tolerance of 0.02 Da and a parent ion tolerance of 7 ppm. Carbamidomethyl on cysteine was specified as the fixed modification. Oxidation on methionine, deamination on asparagine and glutamine, and acetylation on the protein-N term on serine were specified as the variable modifications. Those peptides with no more than 1% FDR (false discovery rate) and those proteins with less than 1% FDR and containing at least 1 unique peptide were filtered for further analysis.

### 4.9. Alignment and Homology Modeling

We used the SWISS-Model and thread-based LOMETS on I-TASSER to predict three-dimensional structural models for putative toxin peptides based on their primary amino acid sequences [87,88]. In the SWISS-Model server, homologous sequences with over 50% sequence identity were used as examined templates.

## 5. Conclusions

In summary, this study reports the first integrated omics analysis to characterize the venom composition of tentacles and column for the sea anemone *H. magnifica*, which will help deepen our understanding of cnidarian toxins, thereby supporting the in-depth development of valuable marine drugs. A total of 728 protein and peptide sequences were identified in the transcriptomes, and 101 sequences were confirmed by proteomics to exist in the venom of tentacles and the column, most of which can be classified as β-defensin peptides, ShK-like peptides, and Kunitz-type peptides. These peptides were then used for homologous modeling and three-dimension structure prediction, and we found that these sea anemone peptides may act on voltage-gated potassium ion channels, voltage-gated sodium ion channels, and nicotinic acetylcholine receptors, potentially becoming leading molecules for innovative marine drugs.

## Data Availability

Raw sequencing reads and Trinity-assembled contigs have been deposited in the NCBI under accession number BioProject PRJNA1023041 (Column: SAMN37638800, Tentacle: SAMN37638799).

## Supplementary Materials

The following supporting information can be downloaded at https://www.mdpi.com/article/10.3390/md22020071/s1. Figure S1: The number of annotated genes in the tentacles transcriptome of *H. magnifica*; Figure S2: The number of annotated genes in the column transcriptome of *H. magnifica*; Figure S3: KEGG pathway annotation of the *H. magnifica* tentacles transcriptome; Figure S4: KOG analysis of the tentacles transcriptome of *H. magnifica*; Figure S5: GO analysis of the tentacles transcriptome of *H. magnifica*; Figure S6: KEGG pathway annotation of the *H. magnifica* column transcriptome; Figure S7: KOG analysis of the column transcriptome of *H. magnifica*; Figure S8: GO analysis of the column transcriptome of *H. magnifica*; Figure S9: Analysis of the tentacles transcriptome of *H. magnifica* through the NCBI Nr database; Figure S10: Analysis of the column transcriptome of *H. magnifica* through the NCBI Nr database; Table S1: Sequencing statistics and assembly summary of the sea anemones transcriptome; Table S2: Numbers of tentacles genes annotated by different databases in *H. magnifica*; Table S3: Number of column genes annotated by different databases in *H. magnifica*; Table S4: Annotation of protein and peptide sequences from tentacles of *H. magnifica*; Table S5: Annotation of protein and peptide sequences from column of *H. magnifica*; Table S6: Sequences identified by proteomics in tentacles and column transcriptome of *H. magnifica*; Table S7: Annotation of *H. magnifica* protein and peptide sequences.
