# Supplementary material for: Venomics Reveals the Venom Complexity of Sea Anemone Heteractis magnifica
Source: Mar Drugs. 2024 Jan 28;22(2):71. doi: 10.3390/md22020071 (PMC10890322; doi:10.3390/md22020071)
Supplement: Supplementary file 1 [file marinedrugs-22-00071-s001.zip › Supplementary Figures.pdf]

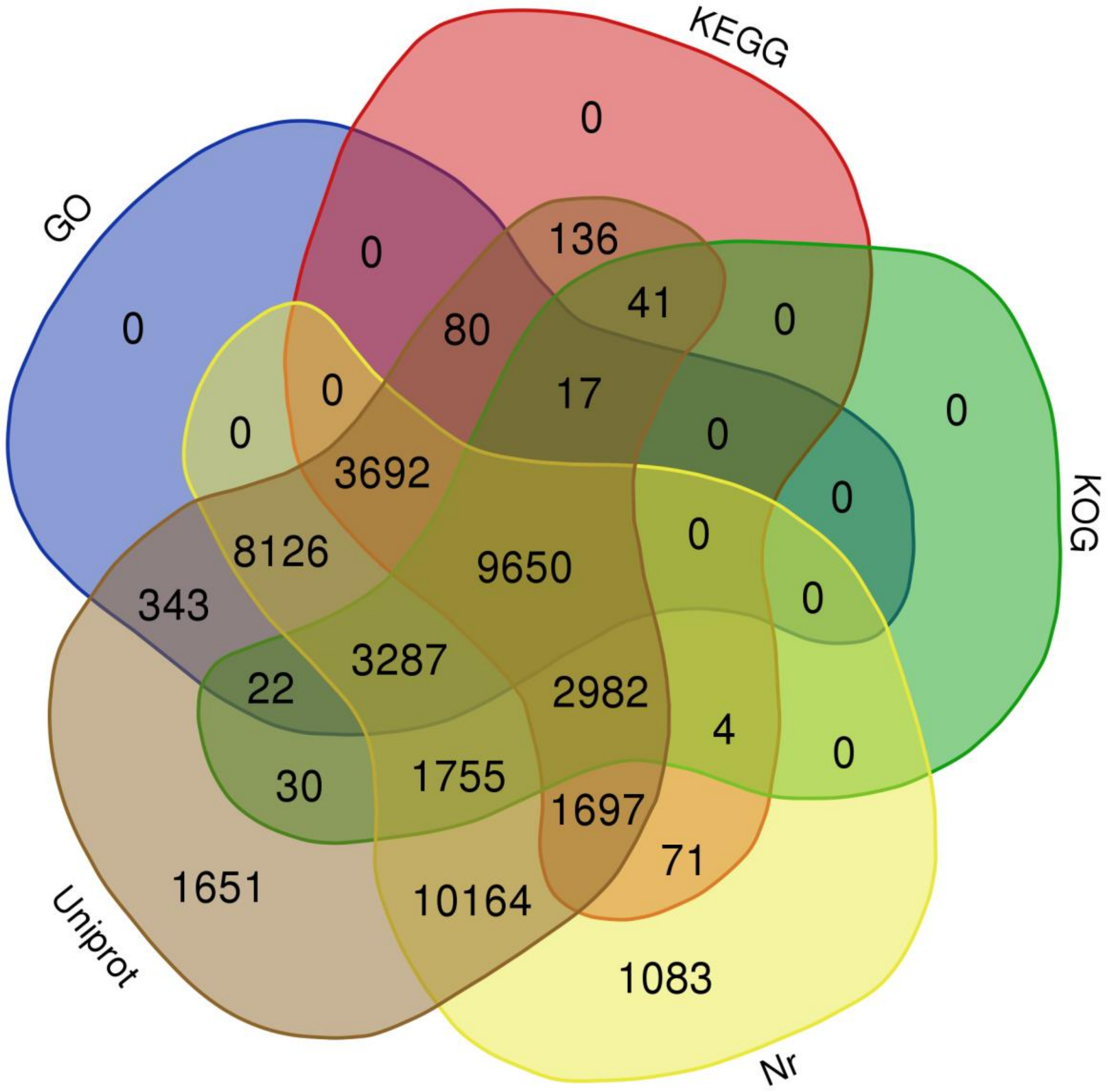

Figure S1. The number of annotated genes in the tentacles transcriptome of *H. magnifica*.

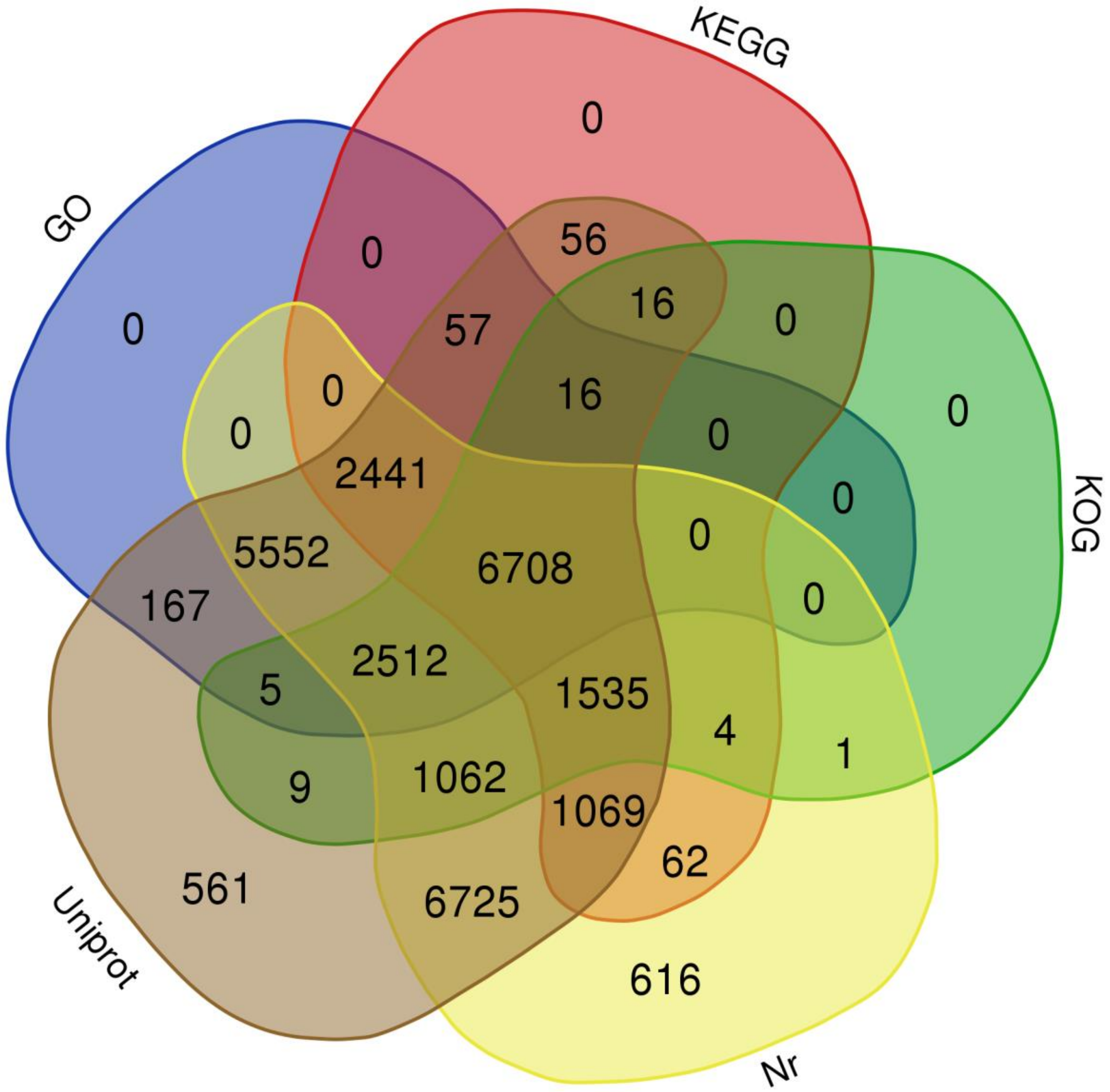

Figure S2. The number of annotated genes in the column transcriptome of *H. magnifica*.

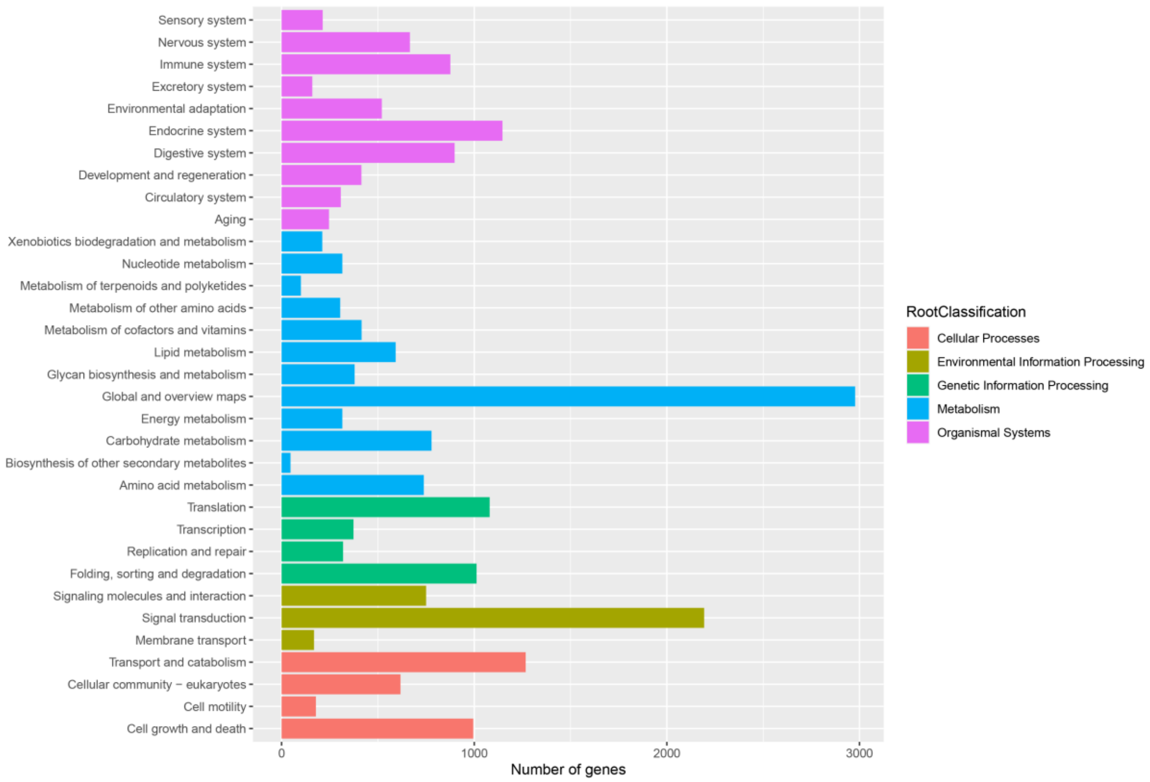

Figure S3. KEGG pathway annotation of the *H. magnifica* tentacles transcriptome.

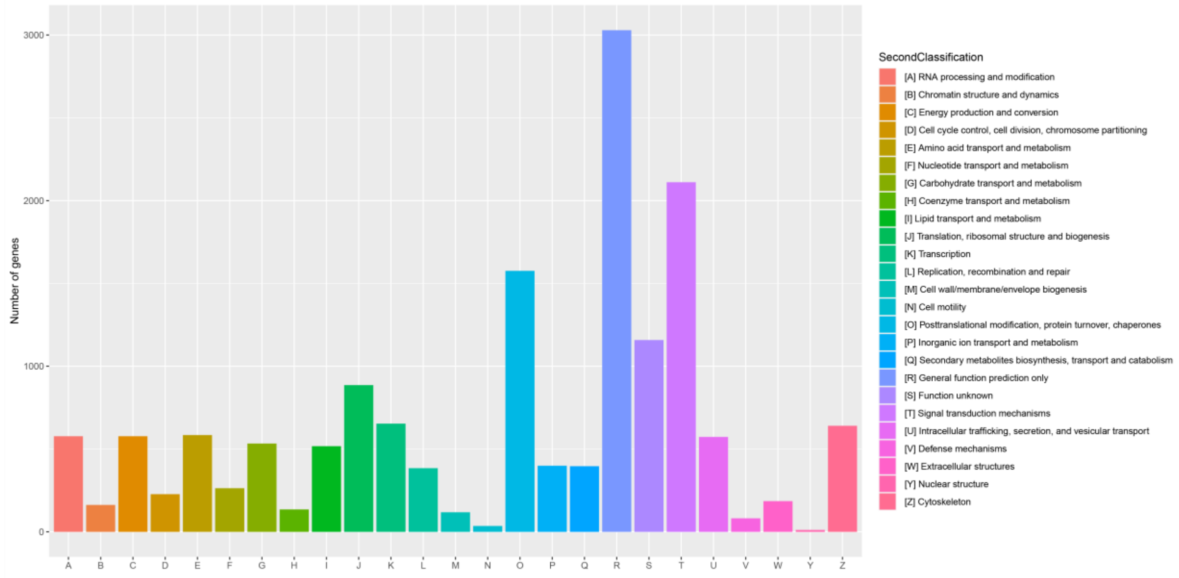

Figure S4. KOG analysis of the tentacles transcriptome of *H. magnifica*.

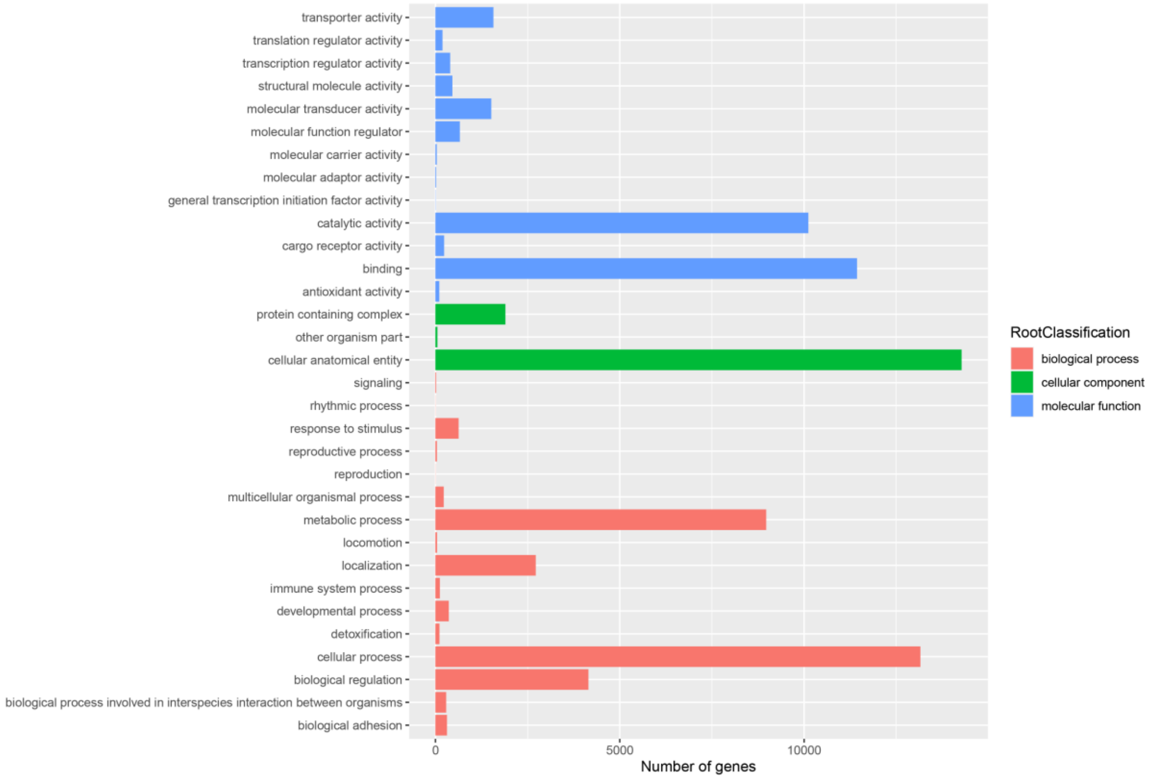

Figure S5. GO analysis of the tentacles transcriptome of *H. magnifica*.

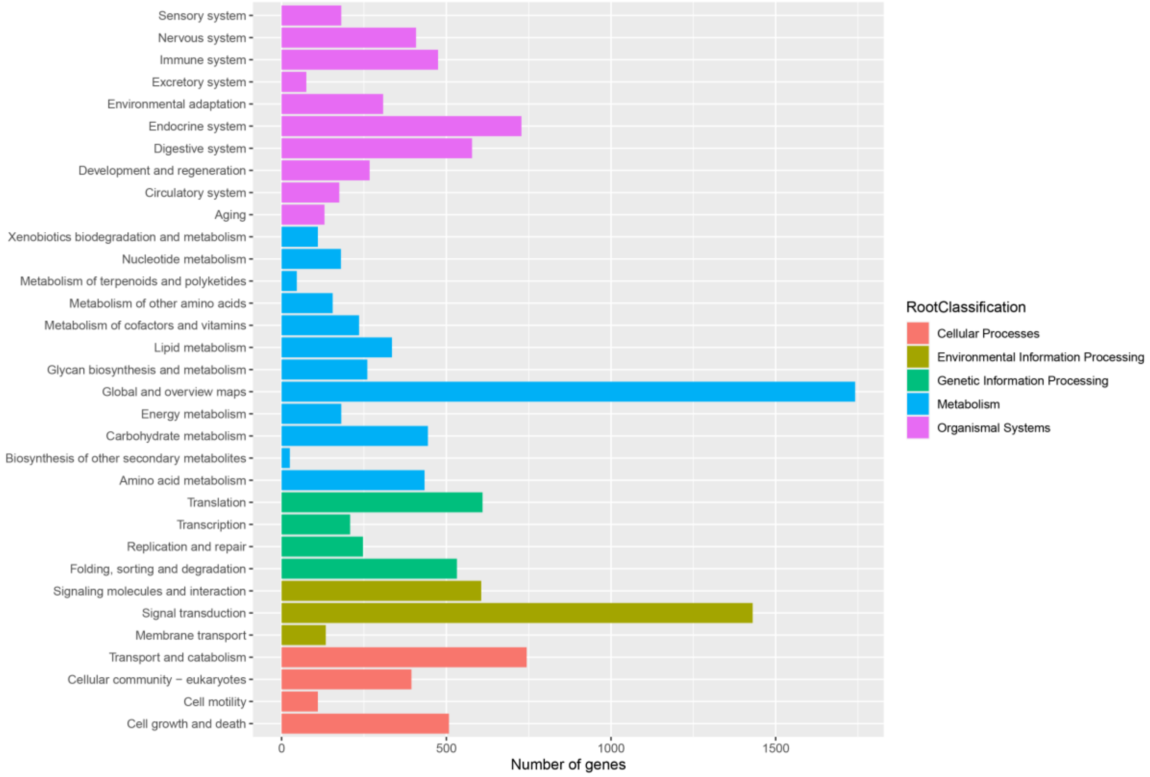

Figure S6. KEGG pathway annotation of the *H. magnifica* column transcriptome.

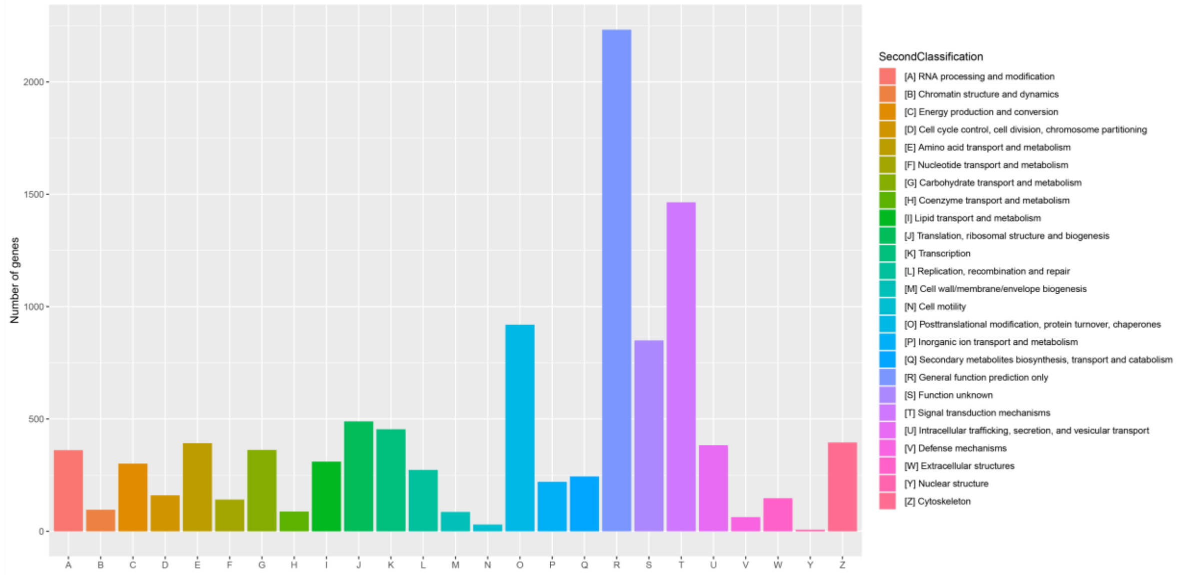

Figure S7. KOG analysis of the column transcriptome of *H. magnifica*.

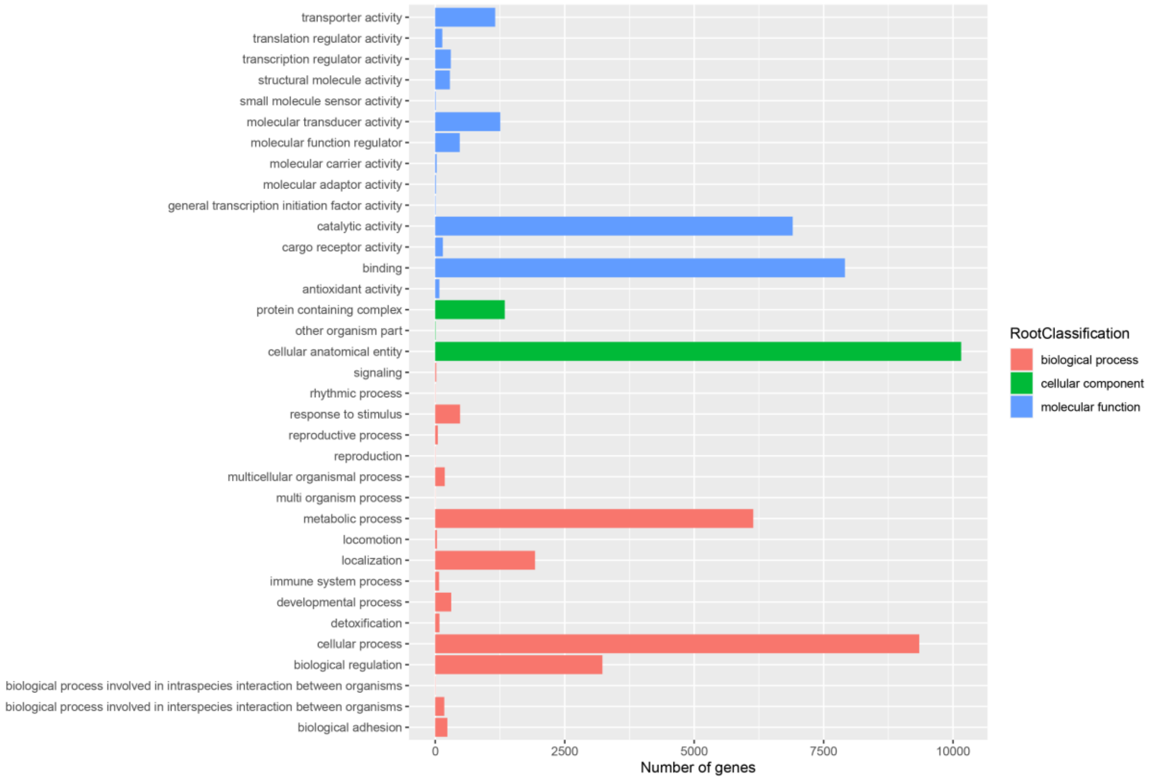

Figure S8. GO analysis of the column transcriptome of *H. magnifica*.

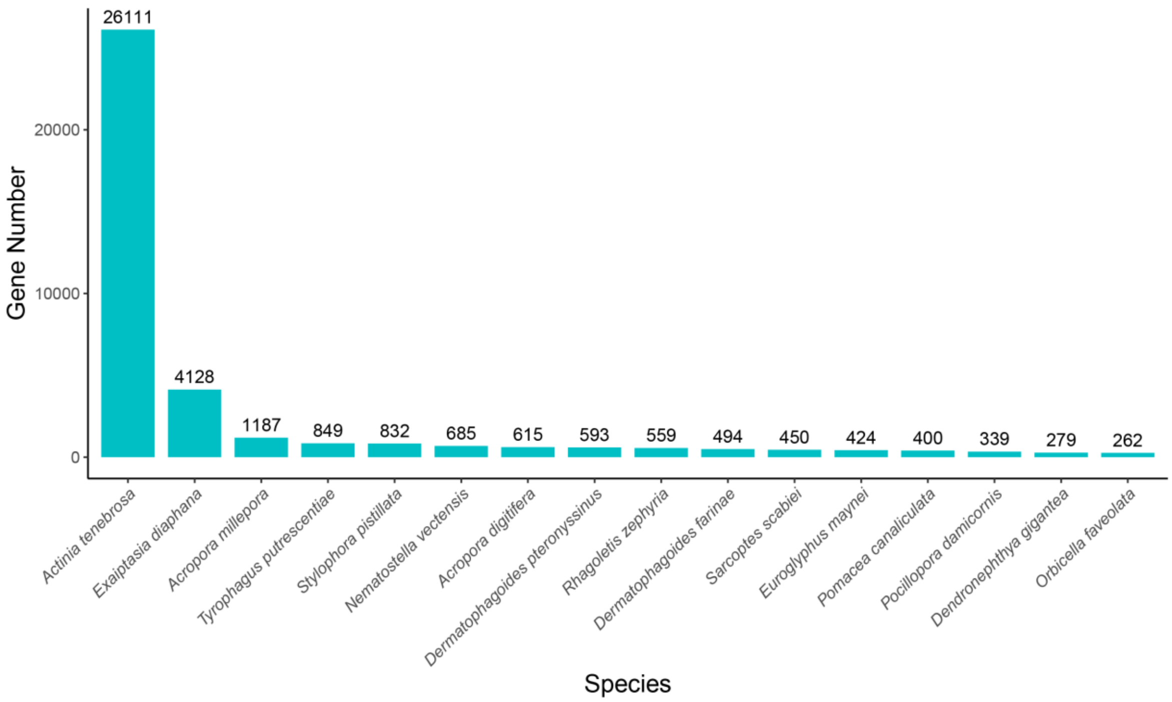

Figure S9. Analysis of the tentacles transcriptome of *H. magnifica* through the NCBI Nr database.

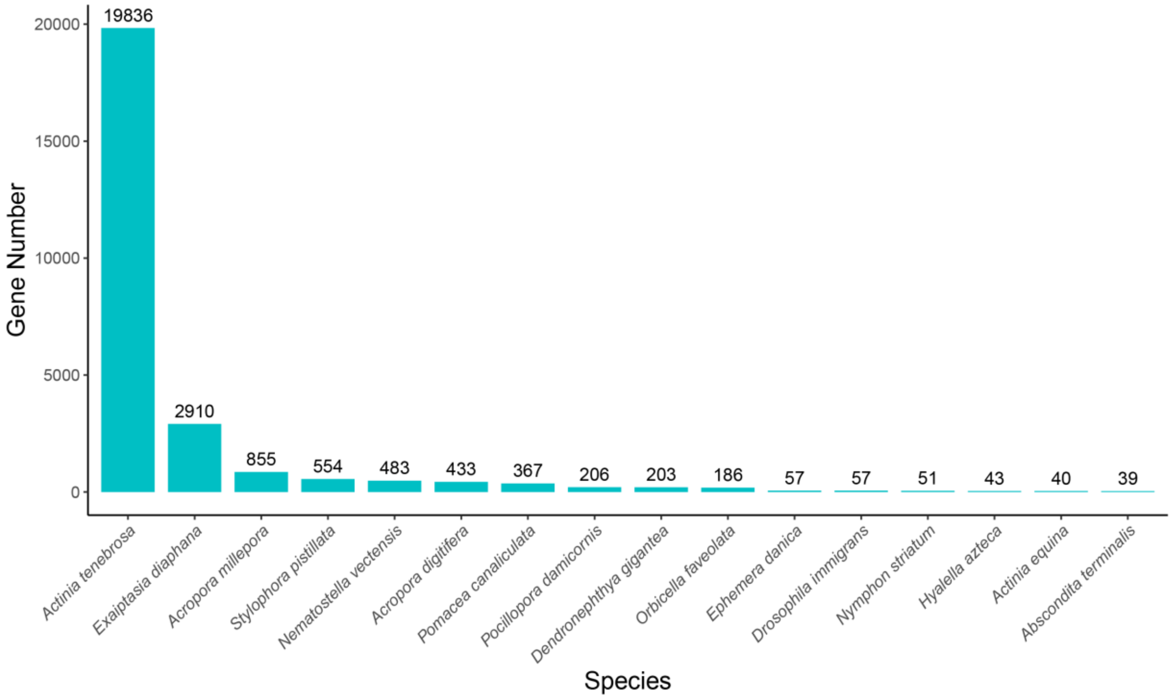

Figure S10. Analysis of the column transcriptome of *H. magnifica* through the NCBI Nr database.
